# Effects of Surface Treatment and Thermocycling on the Shear Bond Strength of Zirconia-Reinforced Lithium Silicate Ceramic

**DOI:** 10.3290/j.jad.b4145161

**Published:** 2023-06-08

**Authors:** Kah Yian Yim, Yew Hin Beh, Chui Ling Goo

**Affiliations:** a Postgraduate, Department of Restorative Dentistry, Faculty of Dentistry, The National University of Malaysia, Jalan Raja Muda Abdul Aziz, Kuala Lumpur, Malaysia. Study design, performed the bond strength test and roughness test, wrote the manuscript.; b Prosthodontist, Department of Restorative Dentistry, Faculty of Dentistry, The National University of Malaysia, Jalan Raja Muda Abdul Aziz, Kuala Lumpur, Malaysia. Study concept, critically reviewed the proposal, data interpretation and data analysis, critcally revised the manuscript critically for important intellectual content.; c Prosthodontist, Department of Restorative Dentistry, Faculty of Dentistry, The National University of Malaysia, Jalan Raja Muda Abdul Aziz, Kuala Lumpur, Malaysia. Study idea, grant application, critcally revised the manuscript critically for important intellectual content.

**Keywords:** zirconia-reinforced lithium-silicate, acid etching, self-etching primer, universal primer, sandblasting, bond strength

## Abstract

**Purpose:**

To investigate the effects of different surface treatments and thermocycling on shear bond strength (SBS) between resin cement and zirconia-reinforced lithium-silicate (ZLS) ceramic.

**Materials and Methods:**

96 ZLS ceramic specimens were randomly allocated to four different surface treatment groups: etch and silane (ES), etch and universal primer (EUP), self-etching primer (SEP), and sandblasting and silane (SS). Standardized composite cylinders were bonded to surface-treated ZLS ceramic, after which SBS was obtained either after 24-h water storage only or with an additional 5000 thermal cycles (TC), resulting in eight subgroups (n = 12). After evaluation of failure mode under a stereomicroscope, representative SEM images were acquired. To examine areal average surface roughness (S_a_), additional ZLS specimens were prepared and randomly allocated to 3 groups: hydrofluoric acid etching, self-etching primer, and sandblasting (n = 10). Supplementary specimens were examined using field-emission scanning electron microscopy (FE-SEM) (n = 2) and atomic force microscopy (AFM) (n = 2) to investigate their surface topographies.

**Results:**

ANOVA showed a statistically significant difference in SBS following different surface treatment protocols after 24-h water storage (p < 0.001). However, TC groups revealed no statistically significant difference in their SBS (p = 0.394). All surface treated groups were significantly affected by TC (p < 0.001), except for the SS group (p = 0.48). S_a_ was significantly influenced by the different surface treatment protocols (p < 0.001).

**Conclusion:**

The ability of self-etching primer to achieve comparable bond strength with a less technique-sensitive approach makes it a favorable alternative to ES for the surface treatment of ZLS ceramics.

All-ceramic indirect restoration materials have emerged as a viable option to restore anterior and posterior teeth because of their outstanding esthetic and mechanical properties.^36^ CAD/CAM with all-ceramic materials is now ubiquitous in the world of modern dentistry. Over the past decade, manufacturers and materials scientists have made great efforts to advance the science of CAD/CAM materials and introduce various CAD/CAM materials onto the market.^8^

A novel material, zirconia-reinforced lithium-silicate (ZLS) ceramic, was recently introduced to fabricate monolithic indirect restorations. The ZLS ceramic is based on a lithium-metasilicate (Li_2_SiO_3_) glass-ceramic that is reinforced with approximately 10% zirconium dioxide (ZrO_2_). The resultant material combines the good mechanical characteristics of zirconia with the positive esthetic appearance of a glass-ceramic.^17^ The ZLS blanks are available in pre-crystalized form; the subsequent crystallisation process helps to convert the ceramic to a fine-grained microstructure of lithium disilicate grains (Li_2_O-ZrO_2_-SiO_2_) with superior mechanical properties. There are generally two systems of ZLS ceramic, marketed as Vita Suprinity PC (Vita Zahnfabrik; Bad Säckingen, Germany) and Celtra Duo (Dentsply Sirona; Konstanz, Germany).^43^ It was found that both of these materials display a similar microstructure of predominantly homogenous glassy matrix consisting of metasilicates and lithium orthophosphates, along with reinforcement of tetragonal zirconia fillers. The grain size of lithium metasilicate is reported to be larger in Celtra Duo than in Vita Suprinity.^17^

ZLS is presumably more similar to lithium disilicate than zirconia. Therefore, it should be susceptible to surface treatment using hydrofluoric acid etching,^27,37^ and thus produce a rough surface for micromechanical retention. Furthermore, ideal ceramic surface morphology after hydrofluoric acid treatment depends on application duration and acid concentration, in addition to simultaneously balancing the surface roughness created while maintaining the strength of the ceramic material.^31,40-42^

With the emergence of other surface treatment options such as self-etching primer (SEP, Monobond Etch & Prime, Ivoclar Vivadent; Schaan, Liechtenstein), the surface treatment and bonding procedure could be combined into a single-step application. Recently, studies have proposed self-etching primers as an alternative to the conventional surface treatment of glass-based ceramic materials.^4,15,26^ Meanwhile, universal adhesive bonding enables uncomplicated bonding processes for glass, oxide ceramics and metal restorations, thanks to its multipurpose nature.^3^ Consequently, while simpler surface treatments are desirable, bond strength reliability of newer ceramic materials (such as ZLS) following conventional surface treatment with hydrofluoric acid and silane has remained ambiguous.

A systematic review by Russo et al^35^ suggested that airborne-particle abrasion is one of the surface treatments of choice, with more evidence suggesting its efficacy for enhancing polycrystalline ceramic materials. Although ZLS contains microstructural zirconium-oxide particle reinforcements, it is still uncertain whether surface treatment with airborne particle abrasion followed by silane application promotes bond strength of this novel material to a level similar to that of zirconia ceramic.

The aim of this study was to investigate the different surface treatment protocols and compare thermocycling effects on shear bond strength (SBS) between resin cement and ZLS ceramic. We also aimed to determine the influence of surface area roughness (S_a_) on bond strength. The first two null hypotheses tested were: (1) different surface treatment protocols and (2) their thermocycling effects do not influence the SBS of the ZLS ceramic. The third null hypothesis is that the surface roughness (S_a_) is not influenced by the different surface treatments of ZLS ceramic.

## Materials and Methods

### Sample Size Calculation

Sample size estimation was calculated using two population means formulae according to Lwanga et al^25^ and Naing.^29^ A minimum sample size of 10 samples per group was determined based on similar research done previously by Kim et al.^23^ An effect size of 5.0 was estimated by considering a level of significance of 0.05 and a power of 80%. After consideration of 10% of drop-out rate, the final sample size selected for each group was 12 samples (n = 12).

### Ceramic Specimen Preparation

**Fig 1:**
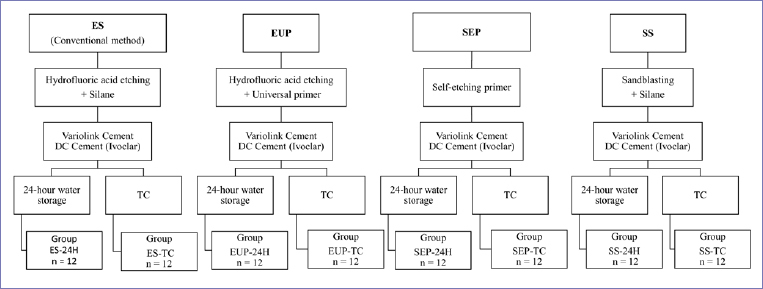
Description of specimen groups subjected to the surface treatment protocol.

First, eight ZLS blocks were sectioned with Isomet 4000 (Linear Precision Saw, Buehler; Lake Bluff, IL, USA) to obtain 96 ZLS specimens. Then, all ceramic specimens were subjected to firing (at an initial temperature of 500°C and terminated at 820°C), following the manufacturerʼs recommendations. Subsequently, the bonding surface of each ceramic specimen was sequentially polished with 600-, 800- and 1200-grit sandpapers under constant cooling using a polishing machine (Ecomet 250, Buehler), after which they were meticulously examined for any gross surface defects under an optical microscope. Material information and descriptions are presented in Table 1. After specimen dimensions had been checked with a digital calliper (Mitutoyo; Kuala Lumpur, Malaysia) to an accuracy of ±0.05 mm, all ZLS ceramic specimens underwent ultrasonic cleaning with distilled water for 10 min and were air dried before continuing the surface treatment procedure. Subsequently, ZLS ceramic specimens (n = 24) were randomly allocated to four different surface treatment groups (Fig 1): etch and silane (ES), etch and universal primer (EUP), self-etching primer (SEP), and sandblasting and silane (SS).

**Table 1:** Material manufacturers, batch numbers, chemical compositions, application modes and procedure after surface treatments

Material manufacturer/batch number	Chemical composition	Application procedure	Procedure after surface treatment
Celtra Duo; Degudent: Hanau, Germany Lot 16005176 REF 5365411025	SiO_2_, Li_2_O, K_2_O, P_2_O_5_, Al_2_O3, ZrO_2_ and CeO_2_, pigments	Firing recommendation: initial temperature at 500°C and termination at 820°C	Polishing in a polishing machine with 600-, 800- and 1200-grit sandpaper under constant cooling
IPS Ceramic Etching Gel; Ivoclar Vivadent REF PEG LOT 181217	9.6% hydrofluoric acid	Application for 30 s with a microbrush	Thorough rinsing with distilled water, then ultrasonic cleaning with the same water for 10 min, followed by air drying
50-μm aluminium oxide REF: H00002	50-μm aluminium oxide	Sandblasting at a standardized distance of 10 mm from the specimen’s surface for 15 s at a pressure of 2 bars	
Silane Coupling Agent; Dentsply Sirona LOT 00077420 REF 607080	Ethanol and acetone	Application with a microbrush for 30 s	Air drying for 60 s
Monobond N, Ivoclar Vivadent REF #642967AN LOT Z00XJM	Alcohol solution containing silane methacrylate, phosphoric acid methacrylate and sulphide methacrylate	Application with a microbrush and left to react for 60 s	Allow to stand for a few seconds, disperse remaining bonding agent with a strong stream of air
Monobond Etch and Prime, Ivoclar Vivadent LOT Z00XFN 6712699	Ammonium polyfluoride and a silane system based on trimethoxypropyl methacrylate, alcohol and water	Application with a microbrush. Then, scrub for 20 s and let react for 40 s	Thorough rinsing with distilled water, followed by air drying
Variolink DC, Ivoclar Vivadent LOT Z01B9H REF #666119WW (Neutral Shade)	Bis-GMA, UDMA, TED-GMA, barium glass, ytterbium trifluoride, Ba-Al-fluorosilicate glass, spheroid mixed oxide, initiators, stabilisers and pigments	Dispensation from an automix syringe at an optimum ratio	Remove the excess cement with a disposable microbrush, followed by light activation with an LED for 40 s on each side along the cement interface
Filtek Z250 XT, 3M Oral Care LOT NE04443 REF 1470A2 (A2 shade)	Resin matrices: bis-GMA, bis-EMA, UDMA, TEG-DMA Filler loading: 60 Vol% silanized zirconia/silica particles	Light curing for 40 s	The resin cylinders should not be additionally polished to conserve the freshness and reactivity (non-converted double bonds) of the composite surface which will subsequently be used for bonding

TEG-DMA: triethylene glycol dimethacrylate; bis-GMA: bisphenol A-glycidyl methacrylate; UDMA: urethane dimethacrylate; bis-EMA: bisphenol A diglycidyl methacrylate ethoxylated.

### Composite Resin Cylinder Preparation

Next, 96 resin composite cylinders with standardized dimensions of 2.96 mm in height and 3 mm in diameter were formed by packing the resin composite (Filtek Z250 XT, 3M Oral Care; St Paul, MN, USA) into a customized metal mold, followed by light curing (Bluephase, Ivoclar Vivadent; Schaan, Liechtenstein) for 40 s. The metal mold was then removed, after which light curing was completed on all surfaces of each composite cylinder. The cylinders were subsequently examined for any composite flashes, which were removed with a sharp blade. Finally, the composite cylinder dimensions were reconfirmed using a digital calliper.

### Surface Treatment Protocol

According to the allocated test group (Fig 1), all surface treatment protocols were conducted following the manufacturer’s instructions (Table 1).

### Bonding Procedure and Thermocycling

After assigning the samples to the individual surface treatment groups, they were bonded onto the prepared composite cylinders using Variolink DC (Ivoclar Vivadent). The preformed composite cylinder was then placed into a customized polymethyl methacrylate plate with a cylindrical cavity 3 mm deep and 3 mm in diameter to create a resin cement thickness of 0.04 mm. Subsequently, finger pressure was applied for 2 min on the center of the ceramic specimen to correctly secure its position prior to transferring it to a customised spring-loaded device with a consistent pressure of 14N to allow complete setting of the cement.^21^ Excess cement was then removed using a disposable microbrush. Light activation using an LED curing lamp (Bluephase, Ivoclar Vivadent) was then performed for 40 s along the bonding interface, and all specimens were subsequently stored in a water bath maintained at 37°C for 24 h. Half of each group’s specimens were tested immediately (Fig 1), and the other half was subjected to 5000 cycles of thermocycling (5°C–55°C) with a dwell time of 30 s and transfer time of 5 s.^19,22^

### Shear Bond Strength (SBS) Test

According to the subgroups, all specimens were subjected to the SBS test using a universal testing machine (Shimadzu Autograph AG-X series; Kyoto, Japan). The shear load was applied at a cross-head speed of 0.5 mm/min with a load cell of 50 kgf until bond failure occurred. Finally, SBS (in MPa) was calculated from the maximum load at failure (N) divided by the bonded surface area (A).

### Failure Mode Analysis

The failure mode of each specimen was evaluated at 30X magnification under a stereomicroscope, followed by classification into three possible failure modes: predominantly adhesive failure, predominantly cohesive failure in ceramic, and predominantly mixed failure. Two randomly selected representative specimens from each group were analyzed using a scanning electron microscope (SEM, Hitachi; Tokyo, Japan) at 40X magnification.

### S_a_ Test and Surface Topography

Additional ceramic specimens were prepared for the contact-profilometer test (n = 10) as above and randomly allocated to three groups – hydrofluoric acid etching, self-etching primer, and sandblasting – to investigate surface roughness (S_a_). Finally, supplementary specimens were analyzed by field-emission scanning electron microscopy (FE-SEM, Zeiss; Oberkochen, Germany) (n = 2) and atomic force microscopy (AFM, Park Systems; Suwon, South Korea) (n = 2) to explore their surface topographies.

### Statistical Analysis

All calculations were conducted using SPSS (v.28.0, IBM; Armonk, NY, USA). Inspection of skewness and kurtosis in addition to the Shapiro-Wilks test were used to test the assumption of normality. Levene’s test was used to check homogeneity. One-way ANOVA and Bonferroni’s post-hoc test were used to analyze the different surface treatment protocols in both the 24-h water storage and TC groups. The paired t-test was then used to analyze the effect of thermocycling effects on SBS. Following different surface treatment methods, one-way ANOVA with Bonferroni’s post-hoc test were finally used for S_a_ assessment. The significance level was set at 5%.

## Results

### SBS Test Results (Table 2)

**Table 2:** Mean (±SD) shear bond strengths in MPa of the different surface treatment methods

Surface treatment method	ES	EUP	SEP	SS
24-h group	44.18^a,A^ (±3.13)	41.28^a,A^ (±2.33)	43.67^a,A^ (±2.95)	34.08^b,A^ (±5.00)
TC group	32.1^a,B^ (±5.36)	32.81^a,B^ (±4.65)	35.43^a,B^ (±4.36)	32.66^a A^ (±5.90)

Similar lowercase superscript letters indicate no significant difference in SBS when comparing different surface treatment protocols for either the 24-h or the thermocycled group (horizontal comparison). Similar uppercase superscript letters indicate no significant difference in SBS when comparing the 24-h to the thermocycled treatment protocols for each surface (vertical comparison). ES: etch and silane; EUP: etch and universal primer; SEP: self-etching primer; SS: sandblasting and silane.

Although ES had the highest SBS among the 24-h water storage groups, SEP yielded the highest SBS after thermocycling. Furthermore, the bond strength was significantly affected by the surface treatment protocol type in the 24-h water storage group (p < 0.001), with Bonferroni’s post-hoc test revealing a significant difference between the SS group and all other three groups (p < 0.001). However, the TC groups revealed no significant difference in SBS following the different surface treatment protocols (p = 0.394).

### Thermocycling Effect (Table 2)

Except for the SS group (p = 0.479), the paired t-test revealed statistically significant differences in SBS when comparing the 24-h water storage to the TC groups for the respective surface treatment types (p < 0.001).

### Mode of Failure

**Fig 2:**
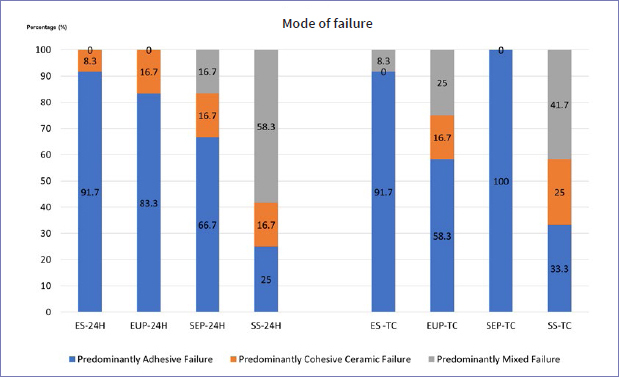
Mode of failure following different surface treatment protocols after 24-h water storage and thermocycling.

**Fig 3:**
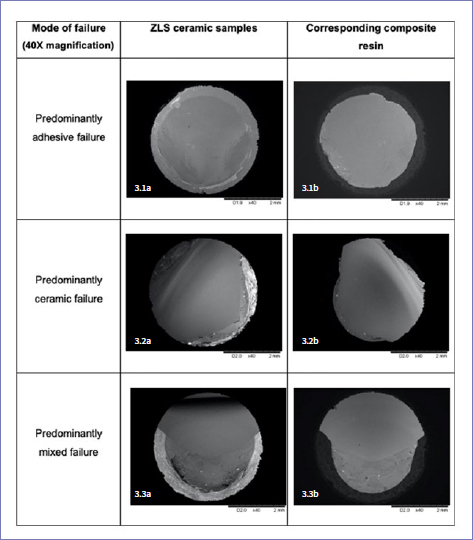
Mode of failure of representative ZLS ceramic samples with their corresponding composite resins (SEM images, 40X magnification).

In both the 24-h water storage and TC groups, predominantly adhesive failure was mainly observed in the ES, EUP and SEP specimens, whereas the SS group mostly presented predominantly mixed failure (Fig 2). Two representative specimens from each group were subsequently analyzed using SEM (40X magnification). The representative SEM images for each type of failure mode are presented in Fig 3.

### S_a_ Test and Surface Topography

**Fig 4:**
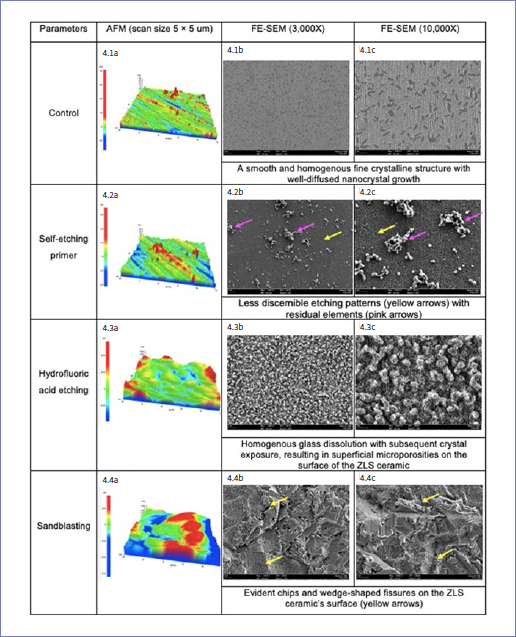
Atomic force microscopy (AFM) and field-emission scanning electron microscopy (FE-SEM) images showing surfaces of ZLS specimens.

One-way ANOVA with Bonferroni’s post-hoc test showed statistically significant differences in S_a_ following different surface treatment protocols (p < 0.05) (Table 3). After sandblasting, while the mean S_a_ was more than twice that of after hydrofluoric acid etching (1.98 vs 0.71 µm, respectively), SEP demonstrated the lowest mean value of 0.09 µm, as measured with the profilometer. The surface topography of each surface treatment method was finally captured by FE-SEM and AFM (Fig 4).

**Table 3:** Mean (±SD) surface roughness (S_a_) recorded using a profilometer after different treatment protocols

Surface treatment method	Mean (±SD) roughness in µm
Sandblasting	1.98 (0.28)^a^
Acid etching	0.71 (0.02)^b^
Self-etching primer	0.09 (0.02)^c^

Different lowercase superscript letters indicate statistically significant differences between the different surface treatment methods (p < 0.05).

## Discussion

This study was designed to investigate the difference in SBS following different surface treatment protocols and whether these protocols are affected by aging (TC). At 24-h water storage, the conventional ES group outperformed the other groups, which concurred with previous studies.^9,20,30,37^ Thus, the first null hypothesis was rejected.

Although the universal adhesive primer in the EUP group contains silane, which is suitable for glass-based ceramic and methacrylate monomers as it includes a functional phosphoric acid group that is effective in establishing bonding to zirconia, the EUP group did not perform as well as the ES and SEP groups. This finding corroborates with a study conducted by Elsayed et al.^18^ Potential interaction between the components in a single bottle is postulated, which may affect their bonding strength, thus resulting in the premature hydrolysis of silane in an acidic environment.^23,28^ Hence, a separate silane is preferable.

All tested groups in this study were significantly affected by the repeated cyclic expansion and contraction stresses posed by thermocycling. This concurs with previous studies,^7,14,28^ finding that thermocycling decreases the bond strength between the predominantly glass-based ceramic and resin cement, even when following the manufacturer’s recommended surface treatment protocol (ES-TC group). Hence, this result led to the rejection of the second null hypothesis. In general, both chemical (acid etching) and mechanical (sandblasting) surface treatments increase the surface roughness, that is, a larger total surface area becomes available for bonding of the ZLS ceramic. However, the long-term bond strength is still susceptible to degradation over time. We suggest that strong hydrofluoric acid etching may initiate microcracks within the material, increasing the susceptibility to crack propagation when the samples are exposed to aging and fatigue stimuli. This may impair the materials’ mechanical strength, especially when over-etched.^31-32,40,42^ This may also explain the occurrence of mixed failure in both the ES-TC and EUP-TC groups.

Conversely, the SEP-TC group exhibited exclusively adhesive failure. This could be due to less severe defects produced by the weaker acidity of ammonium polyfluoride in the SEP-TC group. It is hypothesized that SEP could be a promising alternative surface treatment for ZLS ceramic, since it is least affected by thermal aging and contains a lower risk of over-etching.

On the other hand, it is also interesting to find that the SBS did not differ statistically significantly between the SS-24-h and SS-TC groups (Table 2). It is postulated that the silane and resin cement flowed into the microcracks and deep fissures^10^ that were created following sandblasting, leading to the formation of a thicker hybrid adhesion layer within the ceramic that is not exposed to the external environment. We presume that this led to lower susceptibility to resin degradation for the SS group. Similar findings, in which the SS group was not significantly affected by thermocycling, were also found in a study by Ataol et al.^5^

Furthermore, the phase transformation of zirconia crystals in ZLS may additionally enhance the interlocking microstructure^38,43^ at resin-ceramic interfaces. However, sandblasting is not the surface treatment of choice for ZLS ceramic due to its predominantly glass-based nature, even though it is reinforced with 10% of zirconium dioxide; this agrees with other studies.^1,12,33^ This explains the consistently low bond strength for both SS-24h and SS-TC groups as well as their predominantly mixed and cohesive mode of failure.

Significant differences in surface roughness following different surface treatment protocols led to a rejection of the third null hypothesis. Our study showed that the SS group had the highest surface roughness (Table 3), which was confirmed by the AFM scan (Fig 4.4a) and FE-SEM topographical assessments (Figs 4.4b and 4.4c). The resultant chipping and wedge-shaped fissures following sandblasting may potentially weaken the predominantly glass-based ZLS ceramic. This strongly suggests that the sandblasted ZLS ceramic may crack and fail before the adhesive bond gives way, hence resulting in mainly mixed and cohesive failure for sandblasted specimens. This finding is in line with other studies.^24,33,39^

It has been argued by some researchers^6,13,16^ that the bond strength produced by SEP is inferior to conventional ES surface treatment due to the weaker acidity of ammonium polyfluoride, which promotes a significantly less pronounced etching pattern than does hydrofluoric acid. Accordingly, the lesser penetration extent produced by ammonium polyfluoride in the SEP group was evident in the AFM scan (Fig 4.2a) and FE-SEM images (Fig 4.2b and Fig 4.2c). However, the performance of specimens in the SEP group was similar to that of the conventional method, hydrofluoric acid and silane, which is also in agreement with previous studies^2,4,11,15,20,26,34^ which presented similar outcomes. Therefore, we deduce that milder acid etching with silanation are key factors in ZLS ceramic adhesive bond strength, rather than mechanical surface roughness.

Since aggressive ultrasonic cleaning may affect the chemically bonded silane layer, SEP was the only group that was not subjected to ultrasonic cleaning and was instead thoroughly rinsed with distilled water and air dried after surface treatment, as per the manufacturer’s instructions. In this study, indeterminate residues (pink arrows) were observed in the FE-SEM images (Fig 4.2b and 4.2c). The same was evident in a previous study by El-Damanhoury,^16^ and energy-dispersive x-ray analysis found them to consist of fluorine. However, the clinical significance of these fluorine residues on the bond strength of SEP still requires further exploration.

As a study limitation, while bond strength measurement is common for appraising dental materials’ adhesive properties, these results cannot be directly extrapolated to a clinical environment. The present study was conducted in vitro, so that some clinical conditions could not be exactly reproduced. Moreover, stronger conclusions could have been drawn about the effects of various surface treatments on ZLS if this study had included other ZLS materials, eg, Vita Suprinity (Vita Zahnfabrik) for comparison. Future clinical studies are warranted to validate these findings.

## Conclusion

The ability of self-etching primers to achieve comparable bond strength with a less technique-sensitive approach makes it a viable alternative to etching and silane application for the surface treatment of ZLS ceramics.

## Clinical relevance:

SEP was able to achieve comparable bond strength with a less technique sensitive approach, making it an easier alternative to ES for the surface treatment of ZLS ceramic.
